# The bureaucratic politics of conservation in governing land conflict: A typology of capacities

**DOI:** 10.1016/j.mex.2019.10.022

**Published:** 2019-10-31

**Authors:** Muhammad A.K. Sahide, Micah R. Fisher, Ahmad Maryudi, Grace Yee Wong, Supratman Supratman, Syamsu Alam

**Affiliations:** a Faculty of Forestry, Universitas Hasanuddin, Makassar, Indonesia; b Faculty of Forestry, Universitas Gadjah Mada, Yogyakarta, Indonesia; c Stockholm Resilience Centre, Stockholm University, Sweden

**Keywords:** Conservation bureaucratic politics capacities on governing conflict, Political capacities, Qualitative research, Bureaucratic politics, Conflict governance, Conservation politics

## Abstract

Recent land management policies around the world have experienced a broader political push to resolve forest and land tenure conflict through agrarian reform policy. As a result, conservation bureaucracies are responding with both formal and informal interventions to acknowledge the role of people in forests. In this methods paper, we provide a closer examination of the ways that conservation bureaucracies apply their political capacity in negotiating forest and land tenure conflicts. Our proposed method measures both the capacity and actions of conservation bureaucracies, combining formal dimensions (such as of legal status, budget availability, and the type of organization unit) with informal dimensions (including ways of gaining authority, donors and funding, and trust). The framing is rooted in theories of bureaucratic politics, and while culled from rich empirical experiences from Indonesia, the proposed method is also applicable in examining bureaucratic politics in other natural resource governance contexts.

• We develop a method rooted in bureaucratic politics to measure the capacities of conservation agencies to manage forest land tenure conflict
• The proposed typology guides forest and land use policy researchers to incorporate emergent governance issues such as land tenure reform into their assessments of changing conservation bureaucracies
• The can be adapted for examination of bureaucratic capacities and actions in other contested natural resource contexts

## Graphical abstract

**Figure:**
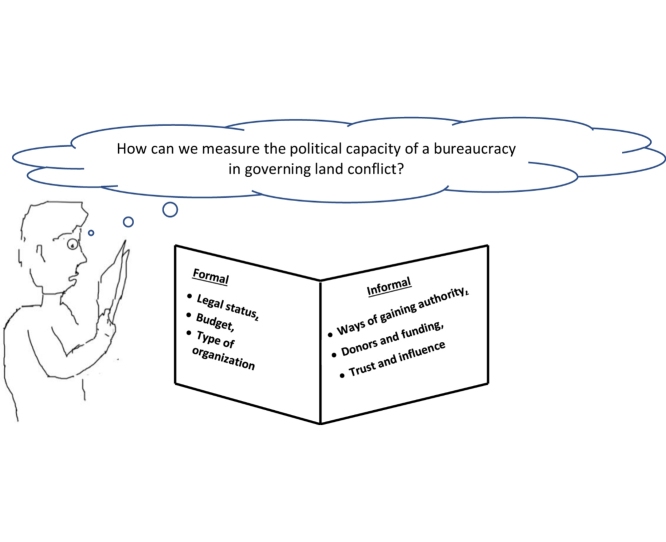

**Specification Table**

**Table:** 

Subject Area:	*Social Sciences*
More specific subject area:	*Forest policy and governance*
Method name:	*Conservation bureaucratic politics capacities on governing conflict*
Name and reference of original method:	*Not applicable*
Resource availability:	*Not applicable*

## Background

Rosati [1] has critiqued the over-utilization of “bureaucratic politics” among scholars for its lack of clear policy application. Hence, several scholars have developed a series of methods to operationalize the meta-theory of bureaucratic politics to a more practical ways, me supporting scholars and practitioners to analyze several land use policy context and their thematic applications. Examples of praxis include the land use visibility conflict typology [2], the framework of deadlock opportunism [3], and a measurement index of the international forest regimes complex relevant for Indonesia [4]. Reflecting upon our longstanding empirical work and observations in forest conservation bureaucracies in Indonesia, we have sought to develop a method for incorporating an emergent and confounding policy dimension involving land tenure conflict and agrarian reform policies.

Conservation bureaucracies are increasingly being tasked with managing and resolving social conflicts around access and claims to protection forests [3,21]. Indeed, this is part of the larger trend of social forestry, agrarian reform, and indigenous politics, discourses that have compellingly argued for a more formally acknowledged role of people in managing forests [20,22]. As such policies are formalized, we seek to do two things. The first is to further provide more policy applicability for theories on bureaucratic politics, particularly as they are related to conservation bureaucracies. The second is to develop a method for addressing new political contestations that help to better understand the political capacity of conservation bureaucracies in governing tenurial conflict. Overall, this is a timely contribution for answering the call for more research on land use conflicts in conservation areas [2], which are facing increasing pressure from internal actors (those involved in land conflicts) [23,24] as well as external actors (such as centralized political actors reshaping the management of conservation areas).

The framework of bureaucratic politics state that ‘bureaucratic politics affect the decision-making of reform policies and managerial tools’ [5]. The conservation bureaucracy, as other bureaucracies have dual tasks, fulfilling formal responsibilities while also pursuing an informal mission. More details on these see the theoretical dimensions are detailed in [16,17]. Overall, the characteristics of the conservation bureaucracy tend to have very strict interpretation of formal rules that prohibit access, envisioning themselves as the last line of defense to protecting forests. For this reason, the face of the conservation bureaucracy is often viewed through the prism of the forest ranger, policing boundaries and denying entry. In some countries, the envisioned role among forests conservation bureaucrats, is gauged by their overall ability to maintain the quantity (not quality) of forests. Conservation bureaucracies therefore, traditionally defined their formal role as the agency that works to maintain biodiversity resources. Meanwhile, informally they also function as any other bureaucracy, seeking to pursue a continued expansion of their role by pursuing goals of expanding staff numbers and, increased access to overall budget portion [9]. Therefore, although the conservation bureaucracy has historically and outwardly described themselves as limiting access to forest resources, they have also reoriented with new mandates for developing formal mechanisms to resolve or manage land and resource conflict. Furthermore, the conservation bureaucracy has also expanded into new realms of capabilities to expand their authority and influence, such as in the form of ecotourism and other sustainable conservation-oriented trade practices [10,11].

## A framework for the bureaucratic politics of conservation: capacities in managing land use conflict

Theoretical investigations of state capacity were conducted by Hendrix [12], who defining the conspicuous quality of the presence and absence of the state in managing conflict. While his framework includes an element of bureaucratic capacity, it lacks the details and tools for measuring broader bureaucratic capacities. The typology that we propose for examining bureaucratic capacities are rooted in the theories of bureaucratic politics, which has treated bureaucracies not as unitary actors, but as actors pursuing formal tasks and informal goals [[6], [7], [8],^16^,^17^]. We take these dual goals, formal and informal, as our main analytic to investigate how a hypothetical local conservation unit conveys their capacities, to manage land use conflict. Bureaucratic capacities are formed by the powers that they wield and the resources on hand, which inherently exist in the role and relations among those in the bureaucracy. These capacities are deployable for achieving particular political agendas for both formal and informal goals (such as formal budget allocation, informal incentives, recognition, awards, and access to more powerful leaders or donors), the large staff quantity and quality, the diverse staff qualification, and others as described in our typology.^1^

For formal capacities, we assess all the formal dimensions of a bureaucracy’s performance. This includes (a) the degree of legal jurisprudence [3,4,13], (b) the level of access to budgets among formal channels [13], and (c) the type of organization, such as its governing hierarchy (national, regional, local) as well as its overall mandate, such as geographic scope and institutional authority (whether administrative or management, and the level of competition with other institutions) [19]. For the informal capacity dimensions, we assess the drivers and motivations behind a bureaucracy’s informal interests in gaining more a) authority, b) informal incentives (such as budgets and support from other avenues), and c) influence or trust. These formal and informal capacity dimensions extends the work of categories established by Rahman and Giessen’s [18] framework that examines informal interests among public actors. They assessed a) responsibility for issues, b) budgets, and c) staff resources. Our framework however, makes a slight distinction in differentiating between formal and informal budgets [15]. Furthermore, the “issues” category in Rahman and Giessen’s [18] framework is too broad to explain what we identify as some distinguishing performance characteristics in the way that a bureaucracy responds to a particular issue like tenurial conflict. Therefore, the notion of what Giessen and Rahman describe as “issues” are diffused within more specific categories distinguished within the formal and informal capacities. For both formal and informal dimensions, we introduce the concept of clients of bureaucracy, which we also interchangeably define as actors [8]. The clients of bureaucracy, therefore, refer to the external actors that cooperate with a bureaucracy.

Hall [14] has strongly suggested that the bureaucratic concept holds greater validity when approaches can be empirically assessed. Our framework builds longstanding empirical work, shaping the plural dimensions of our gradation (scoring) framework on formal and informal capacities. We therefore believe that our proposed framework will travel to numerous other contexts, applicable beyond the singular case of one conservation bureaucracy. To support applications in other context, applicable beyond the singular case of one conservation bureaucracy. To support applications in other context we thus introduce a set of scores to provide a deliberate method in assessing political capacity on managing forest and land use conflict.

### Formal bureaucratic capacities

Within the formal categories listed in Table 1, we ascribe 4 levels of formal capacity dimensions that help to measure the various bureaucratic capacities for forest and land tenure managing conflict. These include the following:

1 Very high formal support, whereby the bureaucracy has a strong legal mandate^2^ and formal structure, supported by a significant budget to resolve or manage conflict, often with the ability to convince actors to offer additional support in handling conflicts
2 High formal support, in which the bureaucracy has a strong formal structure supported by a strong legal mandate. but less support for implementation, such as lacking budget availability, low staff knowledge, inexperience building coalitions, and inadequate skills for conflict mediation
3 Medium formal support, describes a scenario in which the bureaucracy has adequate legal back up^3^ but struggles to interpret laws for implementation, further lacking implementation guidelines hollowing out the intent of the regulations. This scenario also reflects a condition whereby budgets are only available at the administrative level and difficult to mobilize at the ground level
4 Low formal support, poor legal, budgetary and formal structures to back up actions for addressing forest and land tenure conflicts.

**Table 1:** Scoring formal bureaucratic capacities on managing conflict.

Capacity elements	Explanation of formal mandate	Code and score
Legal capacity	All legal intervention are possible and usually occurs when the state has a strong legal mandate and government actors are highly motivated to resolve land tenure conflict	L-4
	The state has a strong legal foundation for resolving tenurial conflict but lack legal instruments for implementing them. But in this condition actors have developed discretionary guidelines based on their motivations for addressing land tenure conflict.	L-3
	The state has a strong legal foundation but do not have a strong legal backup or implementing regulations. However, there may be strong local regulations to support addressing land tenure conflict.	L-2
	The state has a weak mandate, lack implementing instruments, and low incentives to address land tenure conflict. However, there may be limited initiatives at the project or site level that can succeed by citing very broad legal interpretation	L-1
budget capacity	There is a substantial budget available in the central administration government level and also allocated to unit management mandated to formal conflict management responsibilities	B-4
	Budget is only available on the central administration, but there is a hollow budget at the operational level, However, in the scenario implementing units seek to creatively allocate formal budgets for addressing land tenure conflict	B-3
	There is a budget available among operational bureaucracies, but low interest in allocating the budgets to local implementing units because to do so they require new interpretations of nomenclature to assign budgets for managing land tenure conflic	B-2
	Very limited budget initiatives for site level implemntation	B-1
Unit organization capacity	A strong bureaucracy with direct connections to administrative authorities, that has specific and appointed responsibilities for a defined geographic and programmatic mandate (like specific conservation areas), which have a responsibility not only for administering overall programmatic goals but also actively engaged in defining and implementing activities the site level. Such organizational units also have the authority to formalize relations with other institutions and form implementing coalitions	O-4
	Very similar to O-4 but with a key difference that although the geographic area of management may be larger covering a broader land area, these areas also have competing authority from other institutions, and have difficulty in dedicating funds for addressing all the land conflicts across the management area.	O-3
	A lower level bureaucracy (like sub-national authority) mandated to manage a designated area but is not well-connected with higher level administration authority. These institutions also have numerous overlapping mandates, that can range from biodiversity protection to tourism management and other competing interests. Sometimes institutions closer to site-level issues have better relations to address conflict, but sometimes also result in conflict of interests that are difficult to overcome.	O-2
	Similar to O-2 but limited staff available for managing the conflict and less capacity on developing coalition	O-1

### Informal bureaucratic capacities

Within the capacity elements of Table 2, we formulate 4 levels of formal capacity dimensions on measuring bureaucratic capacities for managing forest and land tenure conflict. These include the following:

1 Very high interest: The bureaucracy is interested in expanding and enlarging its power and role, gaining prestige from higher level bureaucracies through their successful prioritization and effective engagement in conflict resolution. These conditions often coincide with recent populist movements seeking to engage rural populations, whereby politicians act as the key actors promoting ‘win-win’ solutions on forest and land tenure conflict, resulting in greater local participation in the overall interests of the conservation bureaucracies.
2 High interest: The bureaucracy would like to gain only their own motive in achieving their interests, usually by creating intensive coalition with relevant actors
3 Medium interest: The bureaucracy endorses external actors, allowing them to facilitate conflict management. They therefore leave it up to external actors to manage conflict and take a passive position, never making important decision, but show that they are paying attention to the conflict
4 Low interest: The bureaucracy has no motivation for managing or resolving land conflict, usually because they have nothing lucrative benefit to gain or lose from conflict management and resolution

**Table 2:** Scoring informal drivers for bureaucratic capacities in managing conflict.

Capacity elements	Explanation of informal mandate	Code and score
Ways of gaining increased authority	Ambitious behavioral from key actors to enlarge their authority through clear action that obtain a positive response from higher level bureaucratic actors, usually supported by effective formal and informal correspondence	A-4
	Ambitious behavioral from key actors to enlarge authority, with effective formal correspondence	A-3
	Ambitious behavioral from key actors to enlarge authority. However, this authority is hidden and communicated only through informal action	A-2
	Willingness to obtain more authority but, there are no effort to gain greater authority	A-1
Gaining informal incentives or non-formal budget	There are substantial incentives gained from their informal actions through non-sate (donor) project or supported by international and national funding related to conflict management	I-4
	There are sufficient budgets and opportunities for proposing and obtaining additional budget from the international donor (or external state bureaucracies) for governing conflict management activities.	I-3
	There is a non-state budget available but requires facilitation by external actors facilitation or coalition to obtain the programs for conflict management	I-2
	No (or little) non-state budget available due to limited information, and access	I-1
Gaining trust from influential external actors	An ability to successfully collaborate and develop a coalition with multiple interests and external actors and focal points (e.g. local communities representatives) from opposing sides or mobilizing neutral party mediators	T-4
	An ability to successfully collaborate and develop a coalition only those that have the same interests of the bureaucracy	T-3
	Actively seeking to build collaboration but unsuccessful in obtaining responses from other actors	T-2
	Open to obtaining support from external actors but little or no effort is made to develop coalitions or uninterested in mobilizing neutral party mediators to connect with others	T-1

## Typology

**Fig. 1:**
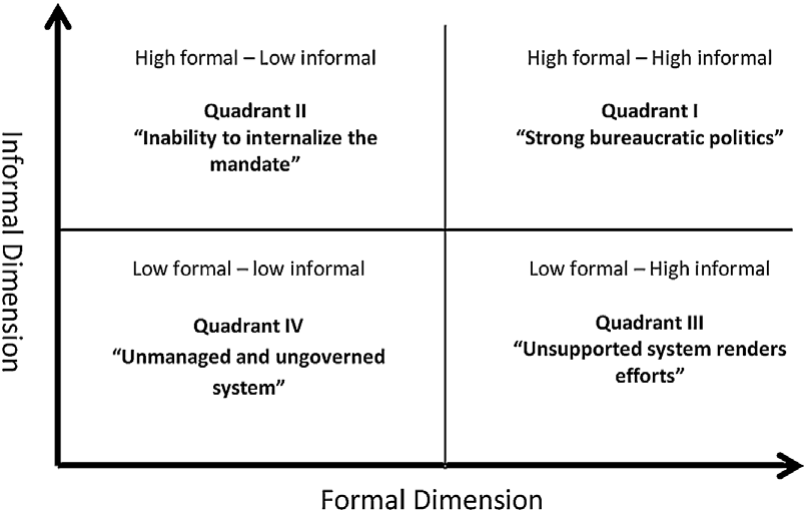
Typology bureaucratic capacities on managing conflict.

The formal and informal capacity elements of the conservation bureaucracy, as described above, come together to provide us with four key capacities that fit together as a broader typology that we describe as four quadrants in Fig. 1

The remark for these four quadrants type is described below

1 High formal - high informal: This is a case of strong bureaucratic politics in managing conflict, and portrays a condition whereby there are enough political capacities to influence opponents in transforming them as partners in a coalition, thus influencing bureaucrats actors, mediators, and advocators
2 Low Formal - high informal: This is a condition whereby the local conservation bureaucracy has a clear motivation to address conflict, and serves to benefit from managing conflict, thus targeting conflict resolution mechanisms or developing conflict governance schemes. However, strict and limited policy support to address conflict can render efforts potentially useless, and worse, may also erode trust between them and local communities
3 High formal - low informal: This is a common situation where centralistic elites among the bureaucracy provide high formal policy support and may provide structural backup for various units to manage conflict. However, though the initiatives may easily pass formal policy channels, they are often unimplemented because they fail to fulfill the motives of the implementing bureaucracy. Alternatively, they fail because of an inability to internalize the formal mandate into real conflict mediation initiatives
4 Low formal - low informal: This is a typical scenario of ungoverned forest and land tenure conflict, unmanaged due to the lack of formal mandate, structure, legislation and low motivation. Indeed, in most cases this condition has historically been the norm, highlighting a lack of initiative or interest in meaningfully engaging in land conflicts.

### Conclusions

This contribution, developing a methods for assessing conservation bureaucracies and their capacity elements for managing forest and land tenure conflict includes two novelties. First, we offer an approach for measuring the multiple dimensions of political capacity among conservation bureaucracies governing forest and land tenure conflicts. Second, we synthesize the broader measurement mechanism into an easily applied typology of political capacity relative to conservation bureaucracies that deal with forest and land tenure conflict. This analytic lens allows for more effectively analyzing the growing trends in policies for agrarian form, and supports future researchers to examine the different formal and informal capacities of the conservation bureaucracies. We believe that empirical applications will help to better pinpoint areas/gaps for improvement in assessing the extent to which forest and land tenure conflicts are likely to be adequately addressed. These methods also provide a foundation for future research by extending their applications with other dependent variables from a range of political dimensions, such as conflict transformation projections, types of conflict coalitions or social movements, visibility of conflicts, and the source/external actors’ motivation of conflicts.

## Declaration of Competing Interest

The authors declare that they have no known competing financial interests or personal relationships that could have appeared to influence the work reported in this paper.

## Footnotes

1 Indonesian context as example, central administration unit like Ministry of Forestry is also mandated as management unit or operational unit under their regional agency (e.g. National Park, Regional Conservation Unit, etc) as consequence that conservation forests is centralized by the law, but still they have a small exception, that Forest Park or *Taman Hutan Raya* could manage by district government, but they are struggling for operationalization back up and budget availability.

## Footnotes

2 Strong legal mandate means that it is back up by the constitution and implicitly mentioned in the law. It reflects securing both basic state legal framework and formal supports and political commitment from the highest levels of political leadership and management within the sector to implement strategies and policies.

3 Enough or adequate legal back up means, it is supported by the sectoral and local regulation (both administration or management regulation).
